# Yawn contagion in bonobos: Another group, another story

**DOI:** 10.1002/ajp.23366

**Published:** 2022-01-31

**Authors:** Ivan Norscia, Marta Caselli, Gabriele De Meo, Giada Cordoni, Jean‐Pascal Guéry, Elisa Demuru

**Affiliations:** ^1^ Department of Life Sciences and Systems Biology University of Torino Torino Italy; ^2^ La Vallée des Singes Romagne France; ^3^ Dynamique Du Langage, CNRS‐UMR 5596 University Lyon 2 Lyon France; ^4^ Equipe de Neuro‐Ethologie Sensorielle, ENES/CRNL, CNRS‐UMR 5292, Inserm UMR S1028 University of Lyon/Saint‐Etienne Saint‐Etienne France

**Keywords:** apes, emotional contagion, Hominini, Pan paniscus, physiological synchronization

## Abstract

In primates, yawn contagion (the yawning response elicited by others' yawn) is variably influenced by individual (e.g., sex, age) and social factors (e.g., familiarity) and possibly linked to interindividual synchronization, coordination, and emotional contagion. Two out of three studies on yawn contagion in bonobos (*Pan paniscus*), found the presence of the phenomenon with mixed results concerning the effect of familiarity and no replication on its modulating factors. To address this puzzling issue, we recorded all occurrences data on yawn contagion in a captive bonobo group (March–June 2021; 18 individuals; La Vallée des Singes, France). Contrary to chimpanzees and humans, the number of triggering yawns increased contagion, possibly owing to a higher stimulus threshold. This aspect may explain the interindividual variability observed in yawn contagion rates. In subjects under weaning, we did not detect yawn contagion and, as it occurs in certain human cohorts, yawn contagion declined with age, possibly due to reduced sensitivity to others. Females responded more than males and elicited more responses from females when showing sexual swelling. As reproductive females are central in bonobo society, our results support the hypothesis that—as in other Hominini—the most influential sex can influence yawn contagion. The relationship quality (measured via grooming/play) did not affect yawn contagion, possibly due to bonobos' xenophilic nature. Overall, this study confirms the presence of yawn contagion in bonobos and introduces new elements on its modulating factors, pointing toward the necessity of cross‐group studies.

## Graphical abstract

Influence of modulating factors on the yawn contagion network, indicated via in‐degree prestige based node size. The different quadrants highlight: (top‐right) age classes (<12 years old: white nodes; 12–30 years old: light grey nodes; over 30: dark grey nodes); (top‐left) sex (males: white nodes; females: gray nodes); (bottom‐right) swelling status within the female network (females without swelling: white nodes; females with swelling: grey nodes). Edge arrows (bottom‐left) indicate the direction of contagion between nodes and go from the trigger to the responder.

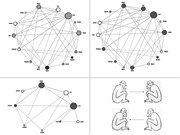

**Abbreviations**
AP: attracted pair
CCT: corrected conciliatory tendency
DP: dispersed pair
Est: estimates
GLMM: generalized linear mixed models
ICT: individual contagion tendency
max: maximum
min: minimum
NDS: Normalized David's Score
NP: neutral pair
PC‐MC: Post Conflict‐Match Control
PY: Post Yawning
PY‐MC: Post Yawning‐Match Control
SE: standard error
VIF: variance inflation factor
vs: versus

## 1 INTRODUCTION

While spontaneous yawning is not dependent on the detection of others' yawns, contagious yawning occurs when the yawn emitted by an individual (hereafter trigger) works as a releasing stimulus (*sensu* Tinbergen & Perdeck, 1950) and induces yawning in another individual (hereafter responder) (Provine, 1989). Spontaneous yawning (or a yawning‐like morphological pattern) is likely a plesiomorphic display because it is present in a wide range of vertebrates (Baenninger, 1987), including human (*Homo sapiens*, Provine, 1986, 2012) and non‐human primates (Anderson, 2020).

Contagious yawning between conspecifics is possibly an apomorphic phenomenon described so far in a limited array of species (Palagi et al., 2020). From an adaptive point of view, yawn contagion can promote synchronization and coordination of activities within social groups (Palagi et al., 2020). Moreover, it can be the expression of interindividual physiological resonance (Prochazkova & Kret, 2017) and possibly emotional contagion, a powerful driver of prosocial behavior (de Waal & Preston, 2017).

Experimental and naturalistic studies on chimpanzees (e.g., Anderson et al., 2004; Campbell & Cox, 2019; Campbell & de Waal, 2011) and humans (e.g., Bartholomew & Cirulli, 2014; Chan & Tseng, 2017; Norscia & Palagi, 2011; Norscia et al., 2021a; Provine, 1986, 1989) have consistently found intraspecific yawn contagion. In bonobos the situation is not as much clear. Amici et al. (2014) examined whether yawning was subject to response facilitation triggered by videorecorded yawns from conspecifics. They found that chimpanzees (14 subjects) but not bonobos (4 subjects) yawned significantly more while or after watching a familiar conspecific yawning on video. On the other hand, on a larger sample (25 subjects), Tan et al. (2017) found that bonobos showed evidence for involuntary, contagious yawning in response to videos of yawning conspecifics. Finally, Demuru and Palagi (2012) also reported yawn contagion in captive bonobos (12 subjects) based on ethological observations under naturalistic conditions. Hence, yawn contagion as a social signal might have been present in the last common ancestor between *Pan* and *Homo*.

Beyond Hominini, it is not possible to associate the emergence of yawn contagion with a single common ancestor. Yawn contagion was not detected in lowland gorillas (*Gorilla gorilla gorilla*; Amici et al., 2014; Palagi et al., 2019) but it was found in orangutans (*Pongo* spp.; van Berlo et al., 2020) which separated earlier from the human line (Groves, 2018). Interestingly, lowland gorillas show low affiliation levels (Palagi et al., 2019) whereas orangutans do not form social groups but orangutans might have been more social in the past (Harrison & Chivers, 2007). In non‐hominid primates, yawn contagion studies show mixed results (cf. geladas, *Theropithecus gelada*, Gallo et al., 2021; Palagi et al., 2009; Tonkean macaque, *Macaca tonkeana*, Palagi & Norscia, 2019; but see: stump‐tailed macaques, *Macaca arctoides*: Paukner & Anderson, 2006; Japanese macaque, *Macaca fuscata*, Palagi & Norscia, 2019). Finally, no evidence of yawn contagion was found in strepsirrhines (*Lemur catta* and *Varecia variegata*, Reddy et al., 2016) even though contagious yawning is present in non primates (Gallup et al., 2015; for review: Palagi et al., 2020). Hence, yawning might have been co‐opted as a communicative signal multiple times over the course of the evolution, in relation to the type of sociality.

When present, yawn contagion in primates usually occurs in the few minutes following the yawning stimulus (hereafter triggering yawn) with a peak in the first minute in Hominini (e.g., humans: Palagi et al., 2014; chimpanzees, *Pan troglodytes*: Campbell & Cox, 2019; and bonobos: Demuru & Palagi, 2012). In humans, perceptual factors may influence the yawning response probability (Massen & Gallup, 2017; Norscia et al., 2020). However, the distance between trigger and responder and/or the number of observed triggering yawns were not found to affect yawn contagion (humans: Norscia & Palagi, 2011; chimpanzees: Campbell & Cox, 2019; geladas: Palagi et al., 2009).

Yawn contagion can be influenced by individual and social factors (Palagi et al., 2020). The age of the responder can affect yawn contagion rates in some cohorts of humans (Anderson & Meno, 2003; Bartholomew & Cirulli, 2014; Helt et al., 2010; Hoogenhout et al., 2013) and chimpanzees (Madsen et al., 2013). No study so far has addressed this issue in bonobos. Moreover, in Hominini the yawning response can vary depending on the sex of the responder or the trigger. For example, women may respond more to others' yawns (Chan & Tseng, 2017; Norscia et al., 2016a, 2016b), although this does not occur in all cohorts (Bartholomew & Cirulli, 2014; Norscia & Palagi, 2011). Moreover, in the *Pan* genus yawning response can vary in relation to the trigger's sex, possibly depending on the social role that each sex has in different species (Demuru & Palagi, 2012; Massen & Gallup, 2017). Finally, yawn contagion was found to be influenced by the level of familiarity between subjects in humans (Norscia & Palagi, 2011; Norscia et al., 2020), chimpanzees (Campbell & de Waal, 2011), and in one out of two groups of bonobos (cf. Demuru & Palagi, 2012; Tan et al., 2017), with highest yawn contagion rates being recorded between particularly familiar subjects.

In sum, two out of the three independent studies on the presence of yawn contagion in bonobos detected the phenomenon (cf. Amici et al., 2014; Demuru & Palagi, 2012; Tan et al., 2017) and yawn contagion was higher between closely bonded (compared to weakly bonded) group mates (Demuru & Palagi, 2012) but not between group mates when compared to non‐group mates (Tan et al., 2017). To better understand the phenomenon, we investigated yawn contagion in yet another group of bonobos. We formulated the following predictions.

*Prediction 1: Presence and distribution of yawn contagion*—Based on previous findings on the presence of yawn contagion in two bonobo groups (Demuru & Palagi, 2012; Tan et al., 2017), we expected to find the phenomenon also in our study group (Prediction 1a). Demuru and Palagi (2012) found the maximum yawn contagion rates in the first minute after the triggering stimulus. Hence, we expected to find a similar result in our study group (Prediction 1b). Because yawn contagion was not found in all bonobos (Amici et al., 2014), we expected to find a high contagion variability across subjects (Prediction 1c).

*Prediction 2: Perceptual factors*—Possibly due to the high visual acuity of anthropoid primates (Fleagle, 2013), the spatial distance from trigger and responder was found to have no effect on yawn contagion in chimpanzees (Campbell & Cox, 2019) and geladas (Palagi et al., 2009). Hence, we expected to find no influence of trigger‐responder distance on yawn contagion in bonobos (Prediction 2a). Moreover, in humans and chimpanzees observing several yawns in a row does not seem to raise the chance of yawn contagion (humans: Norscia & Palagi, 2011; chimpanzees: Campbell & Cox, 2019). Hence, we expected a similar result in bonobos owing to their phylogenetic closeness with humans and chimpanzees (Prediction 2b).

*Prediction 3: Individual and social factors*—In the Hominini, the trigger's rank and sex can have an influence on yawn contagion rates, with individuals responding mostly to men in certain cohorts of humans (for yawns that are heard but not seen; Norscia et al., 2020) and chimpanzees (dominant males especially; Massen & Gallup, 2017) and to females in bonobos (Demuru & Palagi, 2012). While males are central in chimpanzee dominance relationships (Bray et al., 2021; Lewis et al., 2021), in bonobos reproductive females are central in determining group dynamics (e.g., Furuichi, 2011). Hence, we expected that trigger's rank and sex—especially adult females—could play a major role in eliciting the yawning response (Prediction 3a). As concerns the effect of age, no study on bonobos has addressed this factor on yawn contagion so far. However, age appears to have an effect in humans (Anderson & Meno, 2003; Bartholomew a& Cirulli, 2014; Helt et al., 2010) and in chimpanzees (Madsen et al., 2013), with yawn contagion being higher in adults than in immature subjects. In certain cohorts of adult humans, yawning decreases with aging (Bartholomew & Cirulli, 2014). This aspect has not been investigated in chimpanzees. Owing to the phylogenetic closeness of bonobos to humans and chimpanzees (Prüfer et al., 2012), we expected that age might have a similar effect on yawn contagion in our study group (Prediction 3b). In humans and chimpanzees, familiarity between individuals has been reported to increase yawn contagion rates (humans: Norscia & Palagi, 2011; Norscia et al., 2016a; chimpanzees: Campbell & de Waal, 2011). In bonobos, no familiarity effect was found between non‐group members in an experimental setting (using video trials; Tan et al., 2017) but it was found within known subjects in naturalistic conditions, with yawn contagion being highest between closely bonded group mates (Demuru & Palagi, 2012). Thus, we expected to find a positive effect of familiarity on yawn contagion in our bonobo group, observed under naturalistic conditions (Prediction 3c).

## 2 METHODS

### 2.1 Ethics statement

This study is purely observational and non‐manipulative, so no approval was not required from the authors' institutions. This research complies with the American Society of Primatologists Principles for the Ethical Treatment of Non‐Human Primates.

### 2.2 Study site and group

The bonobo study group was housed at La Vallée des Singes (Romagne, France) with no fission‐fusion management. During the day, the subjects could move freely from the indoor enclosure (500 m^2^) to a wooded external island (1 ha), except in case of bad weather (in which case when they were kept indoors. The group was composed of 18 individuals (age range: from 0 to 53 years; mean ± *SE*: 16.722 ± 3.035) including adults (4 males and 7 females; age: ≥12 years); juveniles (2 males and 3 females; age: 6–9 years); one weaning female (4 years old); and one lactating newborn male (4 months when the study started). Maternal kinship was known whereas paternal kinship was not known for all individuals (Full group info: Table S1).

### 2.3 Data collection

Behavioral data were collected via audio‐recordings by two observers (M.C. and G.D.M.) on a daily basis from March to June 2021 (8:30–13:00 or 13:00–17:30; observation hours/individual, mean ± *SE*: 66.30 ± 3.78). Data on grooming, contact sitting and social play (especially present in immatures) were used to determine dyadic affiliation levels and were collected via 10‐min scan sampling (Altmann, 1974). Data on agonistic patterns (including displacements, avoidance, priority on food access, overt aggression, etc.) were gathered via all occurrences sampling method (Altmann, 1974) (full ethogram: Table S2).

**Figure 1:**
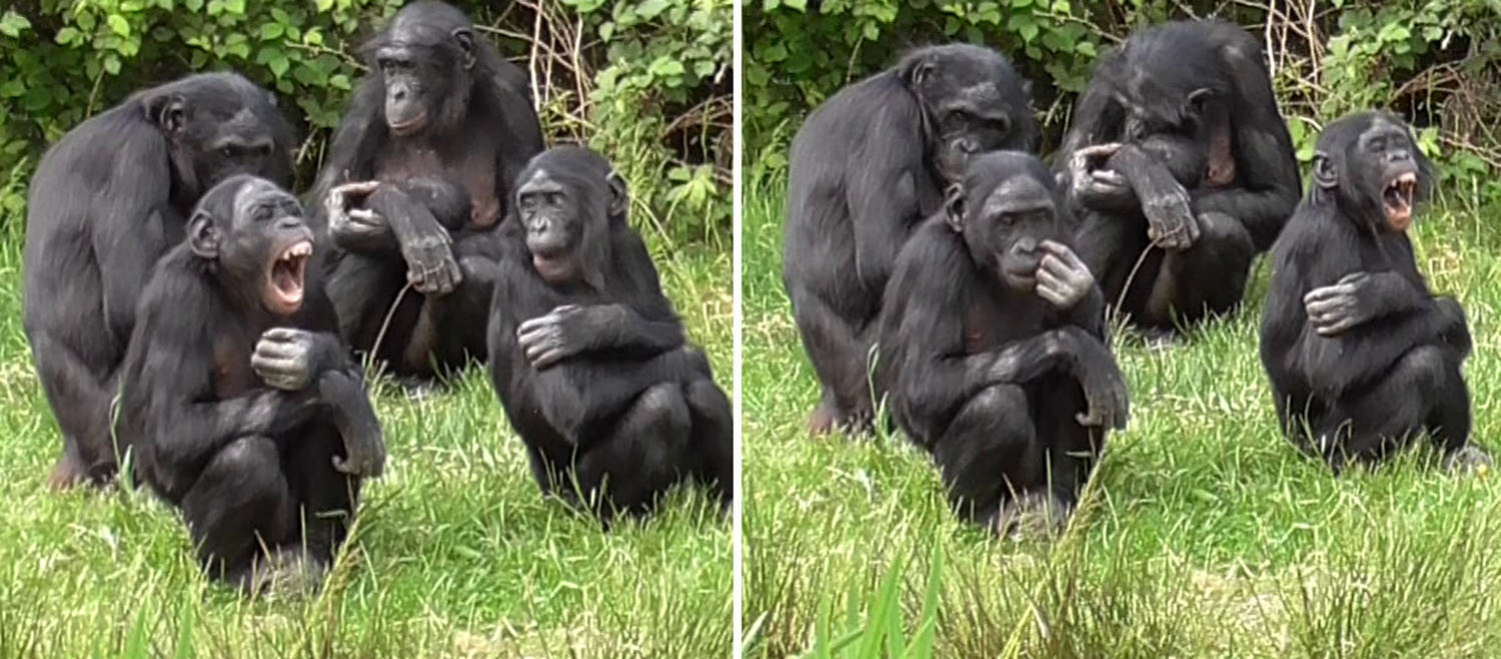
Yawn contagion sequence. Yuli (7yo female) emits a yawn and Lokoro (6yo male) responds after 18 s. Lokoro is sitting within 1 m from Yuli and can see the triggering yawn

Bonobo females show a conspicuous sexual swelling (increased ano‐genital area turgidity) that follows a cycle of roughly 40 days and is not strictly associated with ovulation (Dixson, 1983; Douglas et al., 2016). Data on individual sexual swelling cycle were collected by zookeepers on a specific data sheet, where they indicated whether a female had the swelling cycle (from minimum to maximum size turgidity) or not (menopause: one female; lactation: one female; contraceptive: one female; juveniles females: two). Yawning bouts were collected via the all occurrences sampling method (Altman, 1974) in absence of external perturbing events (e.g., aggression, food distribution; 595 yawns collected in total). The yawning pattern involved mouth opening, with inhalation and a more rapid closing and exhalation (Baenninger, 1997). No yawn was vocalized (via the use of vocal folds). For each yawn (triggering yawn) emitted by a subject, we recorded: (i) identity, sex and age of the yawner (trigger); (ii) identity, sex and age of all the subjects that were visible to the human observer and that could see the triggering yawn (potential responders); (iii) time of the triggering yawn (time of last consecutive yawn if more yawns were emitted in a row); (iv) distance between each potential responder and the last trigger (≤1 m, 1 < distance ≤ 10 m,  >  10 m). A yawn contagion sequence is shown in Figure 1 and Video S1.

Within the 5‐min time window in which a yawning response to a triggering yawn may be observed (Palagi et al., 2009; Provine, 1989), we selected a 3‐min time window to check for the yawning response. We did so as in the fourth minute there is the highest probability of autocorrelation (a yawn performed by a subject at t_0_ increases the probability to have another yawn by the same subject at t_(0+X)_ where X is the increasing unit of time; Kapitány & Nielsen, 2017). To further reduce autocorrelation issues, in case of a yawning chain (i.e., several yawns emitted in a row by the same subject during 3 min, with no other subject yawning), we considered as a response only the first yawn emitted after the last triggering yawn (Gallo et al., 2021). When more than one yawning response occurred from different subjects, the first responder would become a trigger and we noted whether—for each responder—the triggering stimulus came from one or multiple triggers within the fixed time window. For each minute within the 3‐min time window, we recorded the time of each yawning response (if any), so as to determine—*a posteriori* —the minute in which such response occurred (first/second/third) and the identity of the responder. We did not include in the yawning response dataset the subjects (i.e., the potential responders) that had their head rotated by 180° with respect to the trigger or when a physical, sight‐blocking obstacle prevented them from seeing the trigger.

### 2.4 Operational definitions and interobserver reliability

Agonistic encounters, spanning overt aggression and less invasive competitive interactions (e.g., displacements, avoidance, food priority), between individuals were defined as “decided” if a winner and a loser were clearly recognizable and as “undecided” if not. In particular, an individual was considered the loser when they fled, screamed, left the food or the place to the other subject, or emitted submissive vocalizations and/or showed submissive facial expressions (Table S2).

Bonobos showed a strong yawn contagion peak in the first minute after the triggering stimulus in a previous study (Demuru & Palagi, 2012). Thus, we checked for the presence of yawn contagion in each minute of the selected time window. We applied a modified version of the Post‐Conflict/Matched Control (PC‐MC) method, initially designed to check for post‐conflict reunions in animals (de Waal & Yoshihara, 1983) and recently applied to check for grooming contagion (Berthier & Semple, 2018; Ostner et al., 2021) and for the association between spontaneous yawning and behavioral transitions or stressful events (Zannella et al., 2015). In particular, in our case we identified two conditions: (1) Post‐Yawning (PY)—after the last triggering yawn a potential responder was observed in a 3‐min time window to record whether and when (first, second or third PY minute) there was a yawning response; (2) Matched Control (MC)—at the same time (±1 h) as the PY in the first suitable day, under similar social and environmental conditions (e.g. same weather, presence of other subjects) and in the absence of any previous triggering yawn, the same potential responder was observed for three minutes to check whether and when (first, second or third MC minute) yawning occurred.

For each minute, PY‐MC pairs were defined as: (i) attracted (APs) if the yawn occurred in the selected minute in PY and not in MC or if it occurred in PY in a previous minute compared to MC; (ii) dispersed (DPs) if the yawn occurred in the selected minute in MC and not in PY or if it occurred in PY in a following minute compared to MC; (iii) neutral (NPs) if the yawn occurred in the selected minute both in PY and in MC or if it did not occur at all in both conditions.

Based on the method of calculation of the Corrected Conciliatory Tendency (de Waal & Yoshihara, 1983; Veenema et al., 1994) for post‐conflict management, we calculated the Individual Contagion Tendencies (ICTs) as follows: (APs − DPs)/(APs + DPs + NPs). The interobserver reliability between the two data collectors (M.C. and G.D.M.) was calculated via Cohen's k on 10% of the yawning events which they recorded concurrently and independently. Cohen's k was calculated for all the variables considered (yawner identity, possible responder identity, detection condition of the possible responder, distance, yawning response and minute) and was always higher than 0.85 (level of agreement: strong, *sensu* McHugh, 2012).

### 2.5 Statistical elaboration

We determined the individual ranking position and hierarchy steepness based on decided agonistic interactions (ethogram: Table S2) via Normalized David's Scores (NDS; de Vries et al., 2006). NDS were individually assessed via an aggression sociomatrix including the number of decided agonistic encounters/dyad (R ‘steepness’ package; CRAN.R‐project.org/package=steepness). Our study group showed a relatively low steepness (0.425), which indicates a rather shallow hierarchy. Further details provided in appendix S1.

**Figure 2:**
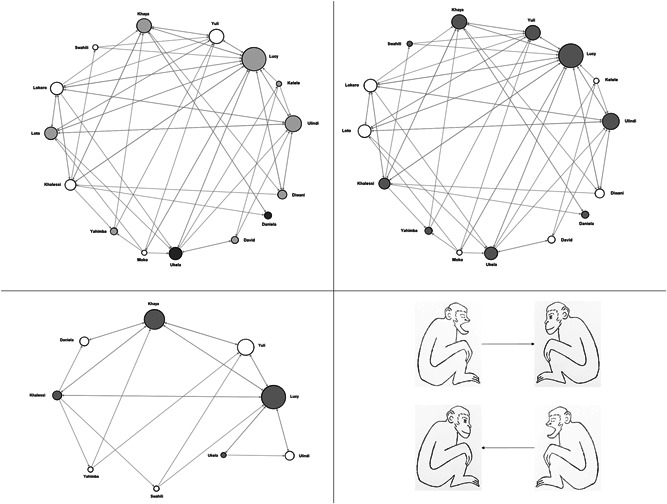
Yawn contagion network. Node size is based on the in‐degree prestige. The different quadrants highlight (top‐left) age classes (<12 years old: white nodes; 12–30 years old: light grey nodes; over 30: dark grey nodes); (top‐right) sex (males: white nodes; females: gray nodes); (bottom‐rleft) swelling status within the female network (females without swelling: white nodes; females with swelling: grey nodes). Edge arrows (bottom‐right) indicate the direction of contagion between nodes and go from the trigger to the responder

Via the freeware Gephi 0.9.2 (www.gephi.org/; dual license CDDL 1.0 and GNU General Public License v3), we obtained the social network of yawn contagion (Figure 2). It includes individuals (nodes) and interindividual connections (directed edges) derived from the number of directional dyadic contagion events (AB if A was the trigger and B the responder; BA if the other way around) normalized over the number of yawns to which the responder was exposed in the 1‐min time‐slot). The node size is based on in‐degree centrality (or prestige) that in our case is the frequency of yawning stimuli received and responded to by a node (sensitivity to contagion; Golbeck, 2013; Saqr et al., 2018). Further details are reported in Appendix S1.

Yawn contagion was never observed in the very few bouts collected on the newborn (which was rarely in a position that allowed reliable yawning detection) and in the 4‐year‐old infant (which would often stay with the mother in non‐observable zones of the enclosure). Therefore, only juveniles (6–7 years old), subadult (9 years old) and adult (≥12 years old) subjects were included in the analyses (*N* = 16 subjects).

To check whether yawn contagion was present (non‐normal data distribution: Kolmogorov–Smirnov, *N*_individuals_ = 15, 0.895 ≤ *Z* ≤ 21.833, *p* < 0.05) we applied the non‐parametric Wilcoxon's pair test to compare the number of attracted versus dispersed pairs at the individual level in each of the three minutes following the triggering stimulus (number of APs > number of DPs). To compare the yawning response level after a triggering stimulus (PY) and in absence of yawning stimuli (MC) we applied a parametric paired *t* test for the first and the second minute (normal data distribution: Kolmogorov–Smirnov, *N*_individuals_ = 15, 0.943 ≤ *Z* ≤ 1.284; *p* = n.s.) and the Wilcoxon's pair test for the third minute (non‐normal distribution: Kolmogorov–Smirnov, *N*_individuals_ = 15, *Z* = 1.833/1.992; *p* < 0.05). Due to the normal distribution of the variables (Kolmogorov–Smirnov, *N*_adults_subtadults_ = 15, *Z* ≥ 0.501, *p* = n.s.), Pearson's bivariate correlation test was used to correlate NDSs and ICTs. To check whether there was a significant variation in the ICTs across subjects, within the group, we applied a one‐sample *t* test (Kolmogorov–Smirnov, *N*_individuals_ = 15, *Z* = 0.710, *p* = n.s.). Based on the PC‐MC method (e.g., Schino et al., 1998), in the previous analysis we included subjects that had at least three PY occasions, so that they could have one pair per type (AP, DP, NP; min PY‐MC pair number for the other subjects: nine).

Because around 85% of the yawning responses occurred in the first minute from the triggering yawn, we verified what factors could affect the yawning response occurring in the 1‐min time slot following the last yawning stimulus from another subject (triggering yawn). To this purpose, we ran two different Generalized Linear Mixed Models (GLMM_1_ and GLMM_2_) on the cases where the yawning stimulus came from a single trigger in the previous minute. In both models, we included the presence/absence of the yawning response as the dependent, binary target variable (presence = 1; absence = 0).

In GLMM_1_ (*N*_cases_ = 344), the following fixed factors were included: (i) triggering yawn number (factor; 1 = one yawn; 2 = two yawns; 3 = more than three yawns); (ii) distance (factor: 1 = individuals within 1 m; 2 = from 1 to 10 m; 3 = more than 10 m); (iii) sex of the trigger and potential responder (factor; M = male; F = female); (iv) age of the trigger and potential responder (numeric; years); (v) trigger and responder rank (numeric; NDS); (vi) affiliation levels (numeric; hourly frequencies with data normalized over the observation time). In GLMM_2_ (*N*_cases_ = 133), run on female dyads only—to check whether the swelling status of trigger and/or responder would affect yawn contagion—the following fixed factors were included: trigger reproductive state and responder swelling status (factor; 0 = without sexual swelling; 1 = with sexual swelling). In both GLMM_1_ and GLMM_2_ the combination between trigger and potential responder's identity (dyad) was included as a random factor.

The GLMMs were fitted in R (R Core Team, 2018; version 3.5.3) by using the function “*glmer*” of the R‐package “*lme4*” (Bates et al., 2015). As a first step we verified if the full model significantly differed from the null model that included the random factors only (Forstmeier & Schielzeth, 2011). The likelihood ratio test (Dobson & Barnett, 2018) was used to test this significance (analysis of variance with argument “*Chisq*”). Subsequently, by using the R‐function “*drop1*,” the *p* values for the individual predictors based on likelihood ratio tests between the full and the null model were calculated (Barr et al., 2013). As the target variables were binomial, a binomial error distribution was used. For significant multinomial predictors, we performed all pairwise comparisons with the Tukey test (Bretz et al., 2010) using a multiple contrast package (*multcomp*). We reported the Bonferroni‐adjusted *p* values, estimate (Est), standard error (*SE*), and *Z* values. We obtained the variance inflation factor (VIF) for the numeric variables of GLMM_1_ via the “*vif*” function in R. All VIF values were between 1 and 2 (min–max range: 1.29–1.48), thus indicating no collinearity. We calculated the effect size via the package “*effectsize*,” function *effectsize* which returns the best effect‐size measure for the provided input GLMM.

## 3 RESULTS

### 3.1 Presence and distribution of yawn contagion

**Figure 3:**
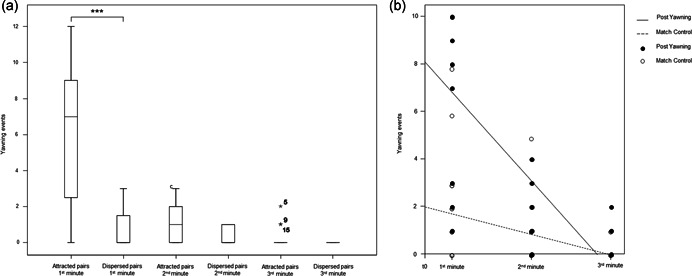
(a) Differences between attracted and dispersed pairs in the 1st, 2nd, and 3rd minute after the yawning stimulus. Solid horizontal lines: medians; box length: interquartile range; thin horizontal lines: observed value range; asterisks: probability level: *p < 0.05; **p < 0.01, ***p < 0.001. (b) Dispersion plot with regression lines showing the decrease of contagious (PY) and spontaneous yawning (MC) in the 3 min following the yawning stimulus

Across the three minutes following a triggering yawn, yawn contagion was present in the first minute, but not in the second and in the third minute. The number of attracted pairs was significantly higher than the number of dispersed pairs in the first minute (Wilcoxon's paired test: *N*_individuals_ = 15, *T* = 3.50, *p* = 0.001), but not in the second minute (nonsignificant trend; Wilcoxon's paired test: *N*_individuals_ = 15, *T* = 12, *p* = 0.053) and in the third minute (Wilcoxon's paired test: *N*_individuals_ = 15, *T* = 0.00, *p* = 0.102) (Figure 3a). Consistently, the level of yawning after a triggering yawn (PY condition) was significantly higher than the level of baseline yawning (MC condition) in the first minute (paired *t* test: *N*_individuals_ = 15, *t* = 3.826, *df* = 14, *p* = 0.002; mean ± *SE*, PY = 7.80 ± 1.93, MC = 1.87 ± 0.61), but not in the following minutes (paired *t* test; 2 min: *N*_individuals_ = 15, *t* = 1.871, *df* = 14, *p* = 0.082; mean ± *SE*, PY = 1.27 ± 0.30, MC = 0.67 ± 0.33; Wilcoxon's paired test: 3 min: *N*_individuals_ = 15, *T* = 1.50, *p* = 0.414; mean ± *SE*, PY = 0.27 ± 0.15, MC = 0.13 ± 0.39; Figure 3b). Within the study group, there was a significant variation in the ICT across individuals (mean ± *SE*: 0.255 ± 0.0423; one‐sample *t* test: *N*_individuals_ = 15, *t* = 5.989, *df* = 14, *p* < 0.001) with one adult male showing no contagion (yawning rate in MC > PY). Figure 2 shows the yawn contagion network and the different parts of the figure highlights different features of the nodes (Figure 2a: age; Figure 2b: sex; Figure 2c: swelling condition).

### 3.2 Variable affecting yawn contagion

**Figure 4:**
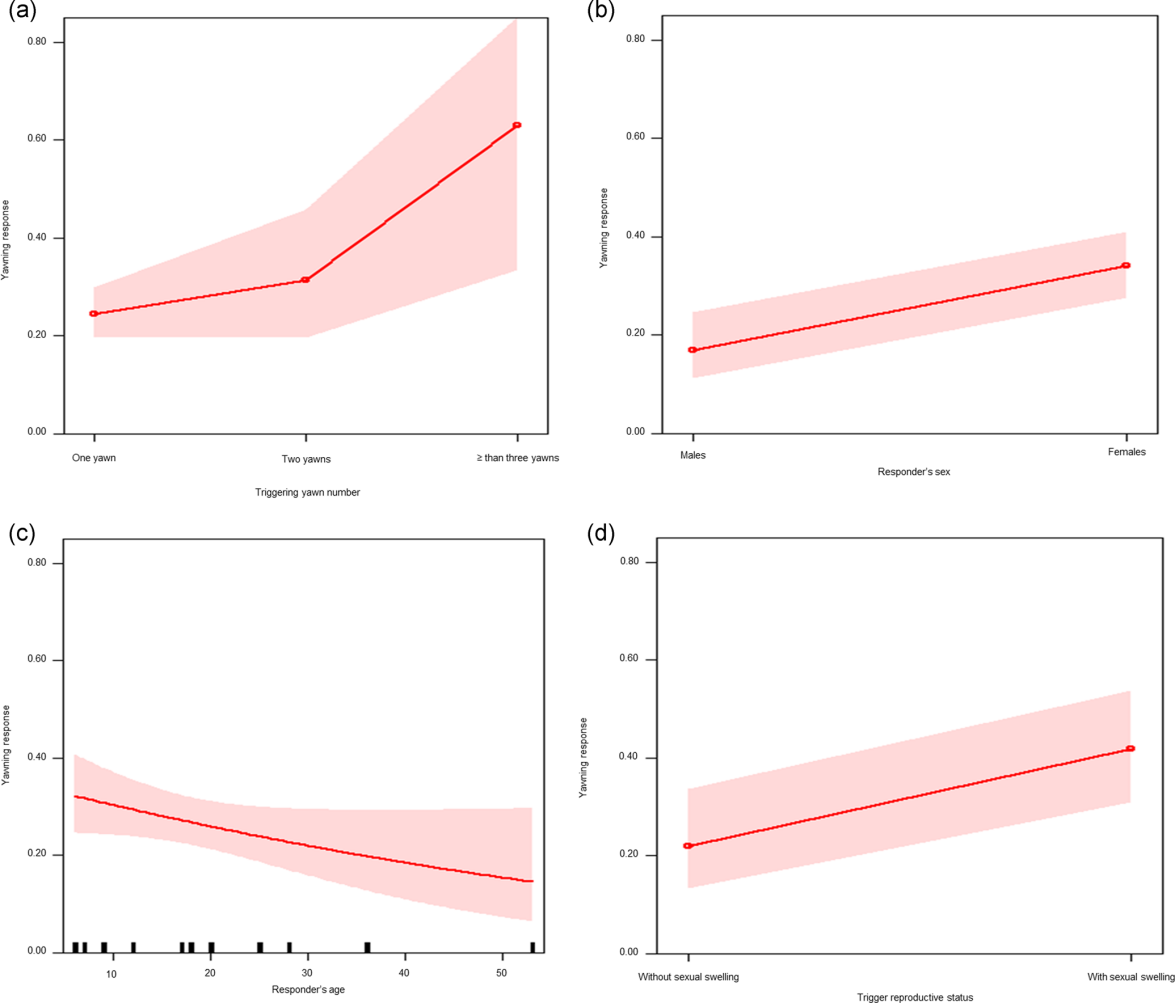
Effect plot of variables having a significant influence on the yawning response. The occurrence of yawning response (Y axis): (a) increases as the “number of trigger yawns” (X axis) increases, (b) varies according to the responder sex (X axis) and is highest in females; (c) decreases as the responder age (X axis) increases; (d) varies according to the trigger sex (X axis) within female‐female dyads and is preferentially triggered by females with swelling. Band represents the confidence interval

The full model (GLMM_1_; target variable: yawning response) including all fixed factors (trigger and responder NDS, trigger yawn number, distance from the trigger, trigger and responder sex and age, social bond) significantly differed from the null model only including the random factor (trigger‐responder identity dyad) (likelihood ratio test: *χ* ^2^ = 26.454, *df* = 11, *p* = 0.006). As at least one predictor had a significant effect on the response, we moved on with a drop1 procedure. We found that the trigger yawn number had a significant effect on the yawning response, which was higher as the number of triggering yawns was ≥ three (Table 1; Figure 4a; Tukey test: 1 vs. 2 yawns, Est = 0.376, *SE* = 0.350, *Z* = 1.076, *p* = 0.514; 1 vs. ≥3 yawns, Est = 1.786, *SE* = 0.636, *Z* = 2.822, *p* = 0.012; 2 vs. ≥3 yawns, Est = 1.409, *SE* = 0.693, *Z* = 2.034, *p* = 0.097). Moreover, the responder's sex had a strong significant effect on the yawning response (Table 1), with females being more likely than males to yawn after perceiving a triggering yawn (Figure 4b). Finally, the responder's age also had a significant effect, with yawn contagion decreasing with age (Table 1 and Figure 4c). No other factors had a significant effect on the target variable (see Table 1 for full results). The second full model (GLMM_2;_ target variable: yawning response) including all fixed factors (trigger reproductive status and responder reproductive status) significantly differed from the null model only including the random factor (trigger‐responder identity dyad) (likelihood ratio test: *χ* ^2^ = 6.668, *df* = 2, *p* = 0.036). As we found that at least one predictor had a significant effect on the response, we moved on with a drop1 procedure. We found that the trigger's swelling status had a strong significant effect on the yawning response (Table 1), with the females showing a swelling cycle eliciting more yawns than those without sexual swelling cycle (i.e., in menopause, lactating or under contraceptives) (Figure 4d).

**Table 1:** Influence of individual, perceptual, social factors (GLMM_1_), and female swelling status (GLMM_2_) on yawn contagion

Predictors	Estimates	SEM	CI_95_	Effect size	*χ* ^2^	*p*
GLMM_1_	*N*_cases_ = 344; full vs. null model: *χ* ^2^ = 26.454; *df* = 11; *p* = 0.006					
(Intercept)^a^	−1.640	0.845	−2.70, −0.86	a	a	a
NDS trigger	−0.017	0.084	−0.35, 0.29	0.03	−0.198	0.843
NDS responder	0.120	0.081	−0.08, 0.60	0.26	1.488	0.137
Trigger yawn number (two yawns)^b^	0.376	0.350	−0.31, 1.06	0.38	1.076	0.282
Trigger yawn number (more than three yawns)^b^	1.786	0.633	0.55, 3.03	1.79	2.822	**0.005**
Affiliation levels	−0.829	1.429	−0.34, 0.18	0.08	−0.580	0.562
Distance (from 1 to 10 m)^b^	0.040	0.382	−0.71, 0.79	0.04	0.103	0.918
Distance (more than 10 m)^b^	0.090	0.533	−0.95, 1.13	0.09	0.169	0.865
Trigger sex (female)^b^	0.021	0.292	−0.55, 0.59	0.02	0.072	0.942
Responder sex (female)^b^	0.945	0.290	0.38, 1.51	0.94	3.263	**0.001**
Trigger age	−0.008	0.016	−0.44, 0.25	0.09	−0.520	0.603
Responder age	−0.038	0.018	−0.82, −0.04	0.43	−2.140	**0.032**
GLMM_2_	*N*_cases_ = 133; full vs. null model: *χ* ^2^ = 6.668; *df* = 2; *p* = 0.036					
(Intercept)^a^	−1.424	0.391	−2.19, −0.66	1.42	a	a
Trigger swelling status (with sexual swelling)^c^	0.947	0.389	0.18, 1.71	0.95	2.434	**0.015**
Responder swelling status (with sexual swelling)^c^	0.248	0.394	−0.52, 1.02	0.25	0.629	0.530

*Note*: Random factor: trigger‐responder dyad. Bold values indicate *p* < 0.05

Abbreviation: CI, confidence interval.

^a^ Not shown as not having a meaningful interpretation.

^b^ These predictors were dummy‐coded, with the reference category as follow: Trigger yawn number: “one yawn”; Social bond: “strong”; Distance: “within one meter”; Trigger sex: “male”; Responder sex: “male.”

^c^ These predictors were dummy‐coded, with the reference category as follow: Trigger swelling status: “without sexual swelling”; Responder swelling status: “without sexual swelling.”

## 4 DISCUSSION

### 4.1 Presence of yawn contagion

Yawn contagion was present in our study group because it was more likely that bonobos yawned after seeing a yawn (PY condition) compared to when they did not observe any previous yawn (MC condition; Prediction 1a supported; Figure 3). Hence, yawn contagion may be present at the population level, as it has been found so far in three different groups (present study; Demuru & Palagi, 2012; Tan et al., 2017). Yawn contagion is also present in different cohorts of other Hominini (chimpanzees: Anderson et al., 2004; Campbell & Cox, 2019; Campbell & de Waal, 2011; humans: e.g., Bartholomew & Cirulli, 2014; Chan & Tseng, 2017; Cordoni et al., 2021; Norscia & Palagi, 2011; Provine, 1989) and—as a form of autonomic contagion—can increase interindividual synchronization and coordination (Casetta et al., 2021; de Waal & Preston, 2017; Prochazkova & Kret, 2017).

We found that the phenomenon was present only in the first minute after the yawn stimulus (Prediction 1b supported), when we detected a significant difference between PY and MC conditions (Figure 3b). On one hand, this result is in line with previous reports showing a peak of yawn contagion in the first minute—compared to following minutes—in bonobos (Demuru & Palagi, 2012) and the other Hominini (chimpanzees: Campbell & Cox, 2019; humans: Palagi et al., 2014). On the other hand, our result introduces an element of novelty because it shows that yawn contagion occurred only (not just maximally) in the first minute (or up to the second minute, if we consider the nonsignificant trend as the basis for further investigation).

In our naturalistic study we found significant variability in the ICTs across subjects (Prediction 2c supported) and one adult male did not show contagion (yawning more in MC than in PY condition). Even though previous studies did not specifically focus on yawn contagion interindividual variation (Amici et al., 2014; Demuru & Palagi, 2012; Tan et al., 2017), Amici et al. (2014) found that in four bonobos yawning was not triggered by video stimuli of yawning conspecifics. Interindividual variability may explain at least in part why contagion is not expressed in all subjects. In healthy humans, 40%–60% of subjects did not show yawn contagion under laboratory conditions (Platek et al., 2003; Provine, 1986, 1989) and susceptibility to others' yawns appears to be stable across contexts (Bartholomew & Cirulli, 2014). Analogously in bonobos, yawn contagion can be context independent (e.g. resting/relaxing vs social tension contexts; Demuru & Palagi, 2012). Future studies on interindividual fluctuations can shed light on within‐population variability.

### 4.2 Perceptual factors affecting yawn contagion

The spatial distance between trigger and responder had no significant effect on yawn contagion (Table 1; Prediction 2a confirmed). Consistently, no influence of trigger‐responder distance was found in chimpanzees (Campbell & Cox, 2019) and geladas (Palagi et al., 2009). This is not surprising because anthropoid primates possess high visual acuity and mainly rely on stereoscopic vision to orient themselves in the world (Fleagle, 2013).

In our study group, yawn contagion probability increased as the number of triggering yawns increased (Prediction 2b not supported; Figure 4a; Table 1). This is in contrast with the situation found in humans and chimpanzees, in which no such effect was found (Campbell & Cox, 2019; Norscia & Palagi, 2011). Interestingly, Norscia et al. (2021b) found that in domestic pigs both trigger‐responder spatial distance and the number of (non‐vocalized) yawning stimuli affected yawn contagion rates possibly due to the scarce visual acuity of the species. It is possible that bonobos—compared to humans—possess a higher yawn contagion threshold and that the yawning response is most likely primed after observing multiple yawns. This possibility may contribute to the interindividual variability observed in bonobo yawn contagion and might point towards possible neurobiological differences in stimulus processing. Future cross‐species studies are necessary to clarify this issue.

### 4.3 Individual and social factors modulating yawn contagion

Compared to males, females were not overall more effective as triggers even though a previous study fount that adult females tended to induce others' yawns more than males (Demuru & Palagi, 2012). This difference may be due to the fact that our female sample included females with and without a swelling cycle, which allowed us to test for this variable (not tested before). We found that females with a swelling cycle elicited more yawning responses from other females compared to females without swelling cycle (Table 1 and Figure 4d). In this respect, Prediction 3a can be at least partially confirmed. Swelling in bonobos is an important communicative signal not just for males but also for females (Demuru et al., 2020) and can contribute to determining their social status by favoring female‐female socio‐sexual interactions and alliances (Furuichi, 2011; Moscovice et al., 2019). Analogously, in chimpanzees ‐ in which males form alliances to control resources (Bray et al., 2021; Lewis et al., 2021)—males seem to be most powerful in eliciting yawn contagion, especially if dominant (Massen & Gallup, 2017). Rank per se had no significant influence on yawn contagion in bonobos possibly due to the high tolerance level of the species (Furuichi, 2011; Hare & Kwetuenda, 2010). Indeed, in our bonobo group hierarchy showed relatively low steepness, which indicates rather shallow hierarchy. Interestingly, females showed the highest yawn contagion rates (Table 1 and Figure 4b), which may related to their central role in bonobo groups. Such a role may require an enhanced sensitivity to social signals, such as yawning, which may favor interindividual synchronization and social cohesion. In humans, an increased yawning response of women has been observed in some cases (Chan & Tseng, 2017; Norscia et al., 2016a) but not in others (Bartholomew & Cirulli, 2014; Norscia & Palagi, 2011). The socio‐cultural influence characterizing different human cohorts makes it hard to single out an unambiguous effect of gender on yawn contagion (Palagi et al., 2020).

We detected no yawn contagion (as responders) in the two infants (aged 4 months and 4 years old) and our statistical analysis on subadults and adults showed that yawn contagion decreased with age (Table 1 and Figure 4c; Prediction 3b confirmed). The responder's age seems to affect yawn contagion also in other Hominini. In chimpanzees yawn contagion was found in adult subjects, but absent in infant subjects (Madsen et al., 2013). In humans, yawn contagion is absent, reduced or differently age‐modulated in infants (Anderson & Meno, 2003; Cordoni et al., 2021; Helt et al., 2010; Millen & Anderson, 2011). In human and non‐human mammals, the increase of yawn contagion with age (especially from the immature phase to adulthood) has been associated with possible maturation of socio‐cognitive abilities and/or neural pathways that decode social cues and with the ontogenetic variation in the ability to identify the internal states of others (Cordoni et al., 2021; Madsen & Persson, 2013; Norscia et al., 2021b).

In certain human cohorts, yawn contagion can decline with age (over 40; Bartholomew & Cirulli, 2014) possibly due to a decreased sensitivity to others' states (Palagi et al., 2020). Yawn contagion—possibly mediated by bottom‐up cognitive processes (Palagi et al., 2020)—might also decrease with age as the result of the increased top‐down mechanisms in emotional processing. Interestingly, in humans aging seems to be associated with a switch from bottom‐up to top‐down processes in emotion appraisal (Petro et al., 2021; Reed & Carstensen, 2012). Further neuroethological studies are necessary to verify these hypotheses.

Finally, the affiliation levels between group mates (a social attachment indicator; Dunbar, 1991) did not affect the likelihood of yawn contagion (Table 1; Prediction 3c not confirmed). Social attachment (informed by affiliation levels, kinship and/or group membership) can increase yawn contagion rates (Palagi et al., 2020). Such effect has been observed in humans (Norscia & Palagi, 2011; Norscia et al., 2016a), chimpanzees (Campbell & de Waal, 2011) and other mammals (e.g., domestic pigs, Norscia et al., 2021b; wolves, Romero et al., 2014). The presence of the so‐called ‘familiarity bias' suggests that emotional contagion may influence the phenomenon of yawn contagion (de Waal & Preston, 2017). In bonobos, the situation is puzzling because no effect of group membership (group vs. non‐group members) was experimentally found in one group (Tan et al., 2017) whereas a positive effect of social bond between group mates was found in another group via a naturalistic approach (affiliation rates and kinship were combined; Demuru & Palagi, 2012). At the very proximate level, the familiarity bias on yawn contagion may be dampened in our study colony by the fact that individuals had been together in the same group—with no fission‐fusion management—for a long time (min–max range: 4–12 years). Affiliation rates occurring in the short term may not reliably inform on long‐term familiarity. At the ultimate level, the xenophilic nature of bonobos (showing affiliation between group residents and non‐residents, high intergroup tolerance and food sharing with strangers; Furuichi, 2011; Idani, 1991; Lucchesi et al., 2020; Tan & Hare, 2013; Tan et al., 2017) may have contributed to reducing the adaptive value of familiarity. The lack of familiarity bias was also found in an opposite situation. Particularly, van Berlo et al. (2020) found the presence of yawn contagion in captive orangutans with no effect of familiarity was detected. Wild orangutans do not live in social groups but show dispersed sociality (with occasional encounters). Here, the effect of familiarity may have a reduced adaptive significance because individuals do not form preferential social bonds or alliances. The opposite cases of bonobos (Demuru & Palagi, 2012; Tan et al., 2017; present study) and orangutans (van Berlo et al., 2020) converge in indicating that the familiarity bias may be related to interindividual cohesion (proximate level) and type of sociality (ultimate level). In contrast with previous reports (Joly‐Mascheroni et al., 2008; Romero et al., 2013; Silva et al., 2012), a meta‐analysis showed that familiarity seems not to affect interspecific yawn contagion between dogs and humans (Neilands et al., 2020). A similar approach could help disentangle the familiarity issue in bonobos, especially if by including data collected with the same methodologies on different colonies. Once again—owing to the differences observed across study groups and sites—we stress the importance of expanding the dataset on yawn contagion to account for intergroup differences and clarify what factors can modulate the phenomenon at the population level.

## AUTHOR CONTRIBUTIONS

**Ivan Norscia**:research resources; **Ivan Norscia, Elisa Demuru, Marta Caselli**: students' training for data collection; **Ivan Norscia**: conceived, wrote the manuscript and analyzed data; **Gabriele De Meo and Marta Caselli**: collected and sorted out data; **Giada Cordoni, Marta Caselli, Elisa Demuru**: revised the manuscript; **Jean‐Pascal Guéry**: provided access to resources and facilities;supported data analyses and conceptualization.

## Supporting information

Supporting information.

Supporting information.

## Data Availability

The study data are available from the corresponding author upon reasonable request.
