# Supplementary material for: Yawn contagion in bonobos: Another group, another story
Source: Am J Primatol. 2022 Jan 31;84(3):e23366. doi: 10.1002/ajp.23366 (PMC9285681; doi:10.1002/ajp.23366)
Supplement: Supplementary file 1 — Supporting information. [file AJP-84-0-s002.docx]

**SUPPORTING INFORMATION**

**Yawn contagion in bonobos: another group, another story**

**Running title: New elements on bonobo yawn contagion**

**Ivan Norscia^a*^, Marta Caselli^a^, Gabriele De Meo^a^, Giada Cordoni^a^, Jean-Pascal Guéry^b^, Elisa Demuru^c,d^**

^a^Department of Life Sciences and Systems Biology, University of Torino, Torino, Italy

^b^La Vallée des Singes, 86700, Romagne, France

^c^Dynamique Du Langage, CNRS-UMR 5596, University Lyon 2, Lyon, France

^d^Equipe de Neuro-Ethologie Sensorielle, ENES/CRNL, CNRS-UMR 5292, Inserm UMR S1028, University of Lyon/Saint-Etienne, Saint-Etienne, France

*Corresponding author: Ivan Norscia^a^, Department of Life Sciences and Systems Biology, University of Torino, via Accademia Albertina 13, 10123, Torino, Italy.

Email: [ivan.norscia@unito.it](about:blank) Tel: +39 011 6704547

**Appendix S1**

**Details on material and methods**

*Hierarchy and ranking position determination*

We determined the bonobo ranking position based on decided conflicts, by using the Normalized David’s Scores (NDS) (de Vries et al., 2006). Via the R ‘steepness’ package (https://CRAN.R-project.org/package=steepness), NDS were individually assessed by using decided agonistic encounters. The individual values (number of decided encounters normalized over the individual observation time) were entered in a sociomatrix. NDSs were calculated on the basis of a dyadic dominance index (Dij) in which the observed proportion of wins (Pij) is corrected for the chance occurrence of the observed outcome. The chance occurrence of the observed outcome is calculated on the basis of a binomial distribution with each subject having an equal chance of winning or losing in every agonistic encounter (de Vries et al., 2006). The hierarchy steepness resulted from the absolute slope of the straight line fitted to the normalized David’s scores plotted against the subjects’ ranks (de Vries et al., 2006).

*Yawn contagion network*

A social network can be modelled as a graph constructed from relational data and can be defined as a set of social entities, such as individuals, with some relationships or interactions between them (Tabassum et al., 2018). Via the freeware Gephi 0.9.2 (www.https://gephi.org/, distributed under the dual license CDDL 1.0 and GNU General Public License v3), we obtained the social network for an ‘immaterial’ inter-individual connection: yawn contagion. The network includes the individuals of the group (nodes) and the interactions between them (edges) and it was derived from frequency of yawn contagion, calculated as follows: number of directional dyadic contagion events (directed edges: AB if A was the trigger and B the responder; BA if the other way around) divided the number of contagion occasions (number of triggering yawns to which the responder was exposed and had the opportunity to respond within 1 minute). We entered the trigger of the behavioral stimulus as source and the responder as target (the nodes). Hence, the connections radiating outwards from a node represent the contagion responses that that specific node induced in other nodes (i.e., the contagion induction influence). The inward connections received by a node correspond to the stimuli that such node received and responded to (sensitivity to contagion).

**References**

de Vries, H., Stevens, J.M., & Vervaecke, H. (2006). Measuring and testing the steepness of dominance hierarchies. *Animal Behaviour*, 71(3), 585-592. https://doi.org/10.1016/j.anbehav.2005.05.015

Tabassum, S., Pereira, F.S., Fernandes, S., & Gama, J. (2018). Social network analysis: An overview. *Wiley Interdisciplinary Reviews: Data Mining and Knowledge Discovery*, 8(5), e1256. https://doi.org/10.1002/widm.1256

**Table S1**

Group composition of the bonobo colony housed at La Vallée des Singes (France)

| Subject | Sex | Age | Mother | Father |
| --- | --- | --- | --- | --- |
| Daniela* | F | 53 | Margrit | Camillo |
| Ukela* | F | 36 | Natalie | Bono |
| Ulindi* | F | 28 | Natalie | Bono |
| Diwani* | M | 25 | Daniela | Masikini |
| David* | M | 20 | Daniela | Kirembo |
| Khaya* | F | 20 | Banya | Keke |
| Lingala | F | 18 | Jill | Mwindu |
| Lucy* | F | 18 | Lorel | Bosondjo |
| Kelele* | M | 17 | Salonga | Ludwig |
| Yahimba* | F | 12 | Kumbuka | Zamba |
| Loto* | M | 12 | Ulindi | Kumo |
| Moko* | M | 9 | Ukela | David |
| Khalessi* | F | 9 | Khaya | David |
| Yuli* | F | 7 | Lucy | Kelele |
| Swahili* | F | 7 | Lingala | Unknown |
| Lokoro* | M | 6 | Ulindi | Unknown |
| Kymia | F | 4 | Ukela | Unknown |
| Yago | M | 0 | Yahimba | Unknown |

* Indicates individuals that were included in the yawn analysis

**Table S2**

Description of the behaviors considered in the present study (Demuru and Palagi, 2012; Kano, 1980; Enomoto, 1990; de Waal, 1988).

| **AFFILIATIVE BEHAVIOR** | **DESCRIPTION** |
| --- | --- |
| **Grooming** | An individual clean another one’s hair both with hands and/or mouth |
| **Reciprocal Grooming** | Two individuals grooming each other |
| **Sit in Contact** | Two or more individuals are sitting in reciprocal contact |
| **Social Play** | Two or more individuals play together. The most common forms of social play are the “rough and tumble”, that can include, for example, play slaps, play bites, play pushes etc., and the “play run”, where one subject chases another one |
| **AGGRESSIVE BEHAVIOR** | **DESCRIPTION** |
| **Avoid** | When an individual avoids interacting with another one, or when it changes its moving direction or goes far away from the latter |
| **Bare Teeth** | Facial expression of fear where all teeth are exposed; usually associated with screaming |
| **Fleeing** | An escape effectuated in an aggressive context |
| **Screaming** | A scream vocalization of fear |
| **Urinate** | An individual urinates for fear in an aggressive context |
| **Defecation** | An individual defecates for fear in an aggressive context |
| **Aggressive Bite** | An individual bites another one |
| **Aggressive Brusque Rush** | An individual jumps on another one |
| **Aggressive Crouching** | A crouching position assumed by an individual who is receiving an aggression, it is displayed to protect itself from the aggressor’s hits |
| **Aggressive Push** | An individual push another one by hands |
| **Aggressive Pull** | An individual pulls another one by hands |
| **Aggressive Slap** | An individual slaps another one by hands |
| **Aggressive Stamping** | An individual jumps on another one with feet together |
| **Charging Display** | It is composed of a series of behaviors (piloerection, run, facial expression, branch dragging, harm swinging, etc.) which generally are displayed by males to threaten other individuals, or to assess their dominance. In bonobo is common even within females |
| **Chase** | An individual chases another one |
| **Kick** | An individual kicks another one |
| **Food Force Claim** | Two individuals get close to food and one of them win in taking it |

Kano, T. (1980). Social behavior of wild pygmy chimpanzees (*Pan paniscus*) of Wamba: A preliminary report. *Journal of Human Evolution*, 9(4), 243-260. doi.org/10.1016/0047-2484(80)90053-6

Enomoto, T. (1990). Social play and sexual behavior of the bonobo (*Pan paniscus*) with special reference to flexibility. *Primates*, 31(4), 469-480. doi.org/10.1007/BF02382531

de Waal, F.B. (1988). The communicative repertoire of captive bonobos (*Pan paniscus*), compared to that of chimpanzees. *Behaviour*, 106(3-4), 183-251. doi.org/10.1163/156853988X00269

**Video S1**

Yawn contagion between Yuli (juvenile female of 7 years old) and Khalessi (subadult female of 9 years old). Yuli emits the yawn at 11:53 a.m. and Khalessi responds after 6s. Khalessi is sitting within 1m from Yuli and can see the triggering yawn.
